# Variable effects of underlying diseases on the prognosis of patients with COVID-19

**DOI:** 10.1371/journal.pone.0254258

**Published:** 2021-07-19

**Authors:** Yong Jun Choi, Ju-Young Park, Hye Sun Lee, Jin Suh, Jeung Yoon Song, Min-Kwang Byun, Jae Hwa Cho, Hyung Jung Kim, Hye Jung Park

**Affiliations:** 1 Department of Internal Medicine, Gangnam Severance Hospital, Yonsei University College of Medicine, Seoul, South Korea; 2 Biostatistics Collaboration Unit, Yonsei University College of Medicine, Seoul, South Korea; Translational Medical Research Institute, Shanghai Public Health Clinical Center, Fudan University, CHINA

## Abstract

Underlying diseases might be risk factors for poor prognosis in patients with coronavirus disease (COVID-19); however, we still do not know whether these diseases are independent factors affecting prognosis, which type of underlying diseases are risk factors, and which type of clinical outcomes are affected. We retrospectively reviewed cohort data from 7,590 de-identified patients with COVID-19 who were diagnosed using severe acute respiratory syndrome-coronavirus-2 RNA polymerase chain reaction test up to May 15, 2020. We used linked-medical claims data provided by the Health Insurance Review and Assessment Service in South Korea. Underlying diseases were identified using the diagnostic codes in the patients’ files from January 1, 2019 to December 31, 2019. The total mortality rate was 3.0% in patients with COVID-19. After adjusting for age, sex, and concomitant chronic conditions, we found that congestive heart failure, chronic pulmonary diseases, diabetes without chronic complications, renal diseases, and malignancy were factors that significantly increased the cost of treatment. Cerebrovascular disease, chronic pulmonary disease, and paralysis were found to be independent factors significant in prolonging hospital stay. Diabetes with chronic complications was independently associated with intensive care unit admission. In addition, underlying congestive heart failure (odds ratio [OR], 1.724; *P* = 0.003), dementia (OR, 1.598; *P* = 0.012), diabetes with and without chronic complications (OR, 1.821; *P* = 0.002 and OR, 1.518; *P* = 0.022, respectively), renal disease (OR, 2.299; *P* = 0.002), and malignancy (OR, 1.529; *P* = 0.039) were significant factors associated with death, even after adjustments. Underlying diseases were significant independent factors of the poor prognosis in patients with COVID-19. The effects were variable according to the type of underlying disease and clinical outcome. Therefore, patients with COVID-19 with underlying diseases should be monitored more closely because they are more at risk of a poor prognosis.

## Introduction

Coronavirus disease-19 (COVID-19), as named by the World Health Organization (WHO), has rapidly spread across the world. The WHO has announced that old age and underlying medical conditions are major risk factors for a poor prognosis in patients with COVID-19, based on simple unadjusted values (accessible at: https://www.who.int/docs/default-source/coronaviruse/situation-reports/20200311-sitrep-51-covid-19.pdf). There are several reasons why underlying diseases may affect prognosis in patients with COVID-19. First, an underlying disease affects the immunity, nutritional status, and general well-being of the patient—factors that play a critical role in overcoming infections, including COVID-19 [1]. Second, the medications prescribed for underlying diseases might limit the use of pharmacologic agents against COVID-19 because of possible drug-to-drug interactions or toxicity from concurrent drug use [2]. Third, underlying diseases are more commonly found in the older population; therefore, COVID-19 can cause lethal outcomes in old age [3].

Recent studies have revealed the prevalence of underlying diseases in patients with COVID-19 and in those who died from the disease [4,5]. Other studies have shown that the mortality rate was increased from the COVID-19 pandemic based on underlying diseases [6]. However, to the best of our knowledge, there have been no studies on 1) whether underlying diseases are independent risk factors for a poor prognosis, even after adjusting for age, sex, and other medical conditions; 2) which underlying diseases significantly affect the prognosis of patients with COVID-19; and 3) which type of COVID-19 clinical outcomes, such as hospital costs and prolonged hospital stay, are affected by underlying diseases. Further epidemiological data are urgently needed to increase the awareness of the association between underlying diseases and COVID-19 and to define which diseases pose a higher risk of poor prognosis.

In this study, we aimed to reveal the various effects of underlying diseases on the prognosis of COVID-19.

## Materials and methods

### Patients and data

The Ministry of Health and Welfare of Korea and Health Insurance Review and Assessment Service (HIRA) of South Korea came up with the world’s first de-identified nationwide COVID-19 medical claims data that included the entire South Korean population (accessible at https://hira-covid19.net). They released the de-identified list of 7,590 patients confirmed as having COVID-19 via an RNA polymerase chain reaction test conducted up until May 15, 2020. All 7,590 patients were included in this study. The diagnostic codes for COVID-19 in the HIRA claims data are as follows: B34.2, coronavirus infection in unspecified area; B97.2, coronavirus as the cause of other diseases in other chapters; U18, tentative designation or emergency use of a new disease in South Korea; U18.1, novel coronavirus infection; and U07.1, COVID-19.

South Korea has a unique national health insurance system that provides insurance for all its citizens. All the medical records and visits are logged in the claims data. HIRA also provided data on the patients with COVID-19’ history of availing medical services for the past 3 years (using finalized claims data, from January 2017 to May 2020). We collected these claims data to analyze the prevalence of underlying disease and clinical outcomes in the patients with COVID-19.

### Underlying disease and Charlson’s Comorbidity Index

We classified 17 underlying diseases (myocardial infarction, congestive heart failure, peripheral vascular disease, cerebrovascular disease, dementia, chronic pulmonary disease, rheumatologic disease, peptic ulcer disease, mild liver disease, diabetes without chronic complications, diabetes with chronic complications, paralysis such as hemiplegia or paraplegia, renal disease, malignancy including leukemia and lymphoma, moderate or severe liver disease, metastatic solid tumor, and acquired immune deficiency syndrome/human immunodeficiency virus) that are known to affect mortality rates and frequently used to calculate Charlson’s Comorbidity Index (CCI) [7]. These underlying diseases were defined by selecting the diagnostic codes that showed up at least once in the patient’s claim data from January 1, 2019 to December 31, 2019; the said codes, used to define the comorbidities were based on widely-accepted guidelines by HIRA (accessible at https://opendata.hira.or.kr/op/opb/selectRfrm.do?rfrmTpCd=&searchCnd=&searchWrd=&sno=11200&pageIndex=1). The CCI, which facilitates the prediction of prognosis and mortality, was calculated as previously described, and according to the HIRA guidelines (S1 Table) [7,8].

### Clinical outcomes

To study the clinical outcomes, we evaluated mortality, admission to intensive care unit (ICU), duration of admission, and total hospital cost for COVID-19 treatment. Mortality and ICU admission were defined based on the associated intervention codes and medical records, respectively. Duration of admission was defined as the length of hospital stay from admission to discharge for a patient with COVID-19. Total hospital cost was defined as the sum of the expenses incurred for all the medical claims records during the treatment period for COVID-19.

### Ethics

This study was approved by the Institutional Review Board of Gangnam Severance Hospital (number: 3-2020-0067). The informed consent requirement was waived due to the minimal risk and retrospective nature of this study using data from the databases.

### Statistical analyses

We used a chi-square test to define significant differences in mortality rates among patients grouped according to underlying diseases. We used linear and logistic regression analyses to define the risk factors for the clinical outcomes. The data were analyzed using SAS Enterprise version 6.1 (SAS Institute Inc., Cary, NC, USA). A *P*<0.05 was used to indicate statistical significance.

## Results

### Clinical characteristics and outcomes of patients with COVID-19

There were 7,590 patients with COVID-19 included in this study. The majority of patients with COVID-19 were in the 20–29 years age group, followed by those in the 50–59- and 60–69-years age groups (24.4%, 19.8%, and 14.0%, respectively). The patients with COVID-19 were predominantly females (59.2%). Among the 17 underlying diseases, chronic pulmonary disease (44.6%), mild liver disease (36.2%), and peptic ulcer disease (32.1%) were the most prevalent.

The total mortality rate was 3.0% in this study. Patients <60 years old showed a relatively low mortality rate (0–0.9%); while those >60 years old showed higher mortality rates (60–69 years old, 3.4%; ≥70 years old, 18.0%). The mortality rate in males (3.9%) was significantly higher than that in females (2.4%; *P*<0.001). The mortality rate in people with underlying diseases (except acquired immune deficiency syndrome) was significantly higher (4.4–19.1%) than that in people without underlying diseases (Table 1).

**Table 1:** Clinical characteristics and outcomes of patients with COVID-19.

Parameters	N (%)	Mortality (%)
**Age (years)**		
0–9	82 (1.1%)	**0 (0.0%)**
10–19	346 (4.6%)	**0 (0.0%)**
20–29	1,855 (24.4%)	**0 (0.0%)**
30–39	776 (10.2%)	**2 (0.3%)**
40–49	1,003 (13.2%)	**1 (0.1%)**
50–59	1,503 (19.8%)	**14 (0.9%)**
60–69	1,061 (14.0%)	**36 (3.4%)**
70+	964 (12.0%)	**174 (18.0%)**
**Sex**		
Male	3,095 (40.8%)	**121 (3.9%)**
Female	4,495 (59.2%)	**106 (2.4%)**
**Charlson’s comorbidity index**		
0	2,029 (26.7%)	
1	1,774 (23.4%)	
2	1,219 (16.1%)	
≥3	2,568 (33.8%)	
**Prevalence of underlying disease**		
Myocardial infarction	199 (2.6%)	**19 (9.6%)**
Congestive heart failure	507 (6.7%)	**85 (16.8%)**
Peripheral vascular disease	1,064 (14.0%)	**90 (8.5%)**
Cerebrovascular disease	787 (10.4%)	**84 (10.7%)**
Dementia	568 (7.5%)	**110 (19.4%)**
Chronic pulmonary disease	3,388 (44.6%)	**151 (4.5%)**
Rheumatologic disease	561 (7.4%)	**25 (4.5%)**
Peptic ulcer disease	2,437 (32.1%)	**108 (4.4%)**
Mild liver disease	2,747 (36.2%)	**132 (4.8%)**
Diabetes without chronic complications	1,775 (23.4%)	**146 (8.2%)**
Diabetes with chronic complications	506 (6.7%)	**70 (13.8%)**
Paralysis (hemiplegia or paraplegia)	145 (1.9%)	**24 (16.6%)**
Renal disease	162 (2.1%)	**31 (19.1%)**
Malignancy	557 (7.3%)	**53 (9.5%)**
Moderate or severe liver disease	26 (0.3%)	**3 (11.5%)**
Metastatic solid tumor	61 (0.8%)	**7 (11.5%)**
Acquired immune deficiency syndrome	7 (0.1%)	1 (14.3%)
**Prognosis**		
Total hospital cost (dollars*)	4,293.4±5,549.1^#^	
Duration of admission (days)	20.1±12.2^#^	
Admission to ICU	215 (2.8%)	
Mortality	227 (3.0%)	
**Total**	**7,590**	**227 (3.0%)**

* 1 dollar = 1,228.40 Korean Won as of June 1, 2020.

^#^ mean±standard deviation.

Text in bold means a statistically significant difference.

COVID-19, coronavirus disease; ICU, intensive care unit.

### Significant factors for mortality

All the variables except that of having acquired immune deficiency syndrome were found to be significant factors that influenced mortality in univariate analysis. After adjusting for other factors, old age (odds ratio [OR], 1.114; 95% confidence interval [CI], 1.097–1.131; *P*<0.001) and male sex (OR for female, 0.385; 95% CI, 0.280–0.529; *P*<0.001) were revealed to be significant risk factors for death. Patients with COVID-19 with underlying congestive heart failure (OR, 1.724; 95% CI, 1.211–2.456; *P* = 0.003), dementia (OR, 1.598; 95% CI, 1.108–2.305; *P* = 0.012), diabetes with or without chronic complications (OR, 1.821; 95% CI, 1.255–2.644; P = 0.002 and OR, 1.518; 95% CI, 1.063–2.169; *P* = 0.022, respectively), renal disease (OR, 2.299; 95% CI, 1.371–3.858; *P* = 0.002), and/or malignancy (OR, 1.529; 95% CI, 1.022–2.257; *P* = 0.039) were significantly at higher risk of death (Table 2 and S1 Fig).

**Table 2:** Significant factors for mortality.

Parameters	Univariate analysis			Multivariate analysis		
	OR	95% CI	*P*-value	OR	95% CI	*P*-value
**Age (years)**	1.127	1.114–1.141	<0.001	**1.114**	**1.097**–**1.131**	**<0.001**
**Sex (female)**	0.594	0.45–0.774	<0.001	**0.385**	**0.280**–**0.529**	**<0.001**
**Prevalence of underlying disease**						
Myocardial infarction	3.645	2.228–5.965	<0.001	0.616	0.342–1.108	0.106
Congestive heart failure	9.846	7.395–13.108	<0.001	**1.724**	**1.211**–**2.456**	**0.003**
Peripheral vascular disease	4.310	3.276–5.670	<0.001	1.063	0.761–1.484	0.721
Cerebrovascular disease	5.566	4.205–7.368	<0.001	0.779	0.540–1.125	0.184
Dementia	14.174	10.745–18.697	<0.001	**1.598**	**1.108**–**2.305**	**0.012**
Chronic pulmonary disease	2.532	1.915–3.349	<0.001	1.208	0.867–1.684	0.264
Rheumatologic disease	1.576	1.031–2.411	0.036	1.144	0.700–1.870	0.592
Peptic ulcer disease	1.962	1.505–2.557	<0.001	0.831	0.604–1.143	0.255
Mild liver disease	2.523	1.930–3.298	<0.001	0.856	0.610–1.200	0.366
Diabetes without chronic complications	6.345	4.810–8.371	<0.001	**1.518**	**1.063**–**2.169**	**0.022**
Diabetes with chronic complications	7.084	5.259–9.542	<0.001	**1.821**	**1.255**–**2.644**	**0.002**
Paralysis (hemiplegia or paraplegia)	7.076	4.469–11.205	<0.001	1.703	0.980–2.959	0.059
Renal disease	8.731	5.758–13.241	<0.001	**2.299**	**1.371**–**3.858**	**0.002**
Malignancy	4.146	3.009–5.713	<0.001	**1.520**	**1.022**–**2.257**	**0.039**
Moderate or severe liver disease	4.274	1.274–14.339	<0.001	1.482	0.328–6.694	0.609
Metastatic solid tumor	4.307	1.938–9.572	<0.001	2.112	0.787–5.667	0.138
Acquired immune deficiency syndrome	5.426	0.651–45.253	0.118	7.080	0.462–108.575	0.160

The text in bold means statistically significant difference in multivariate analysis.

OR, odds ratio; CI, confidence interval.

### Significant factors for ICU admission

Univariate analysis showed that age, sex, and underlying diseases—except moderate or severe liver diseases and acquired immune deficiency syndrome—were significant factors that influenced ICU admission. Older patients (OR, 1.050; 95% CI, 1.039–1.061; *P*<0.001), males (OR for female, 0.518; 95% CI, 0.389–0.689; *P*<0.001), and patients with diabetes with chronic complications (OR, 1.811; 95% CI, 1.241–2.642; *P* = 0.002) were significantly at risk of ICU admission in multivariate analysis (Table 3).

**Table 3:** Significant factors for ICU admission.

Parameters	Univariate analysis			Multivariate analysis		
	OR	95% CI	*P*-value	OR	95% CI	*P*-value
**Age (years)**	1.056	1.048–1.065	<0.001	**1.050**	**1.039**–**1.061**	**<0.001**
**Sex (female)**	0.567	0.432–0.745	<0.001	**0.518**	**0.389**–**0.689**	**<0.001**
**Prevalence of underlying disease**						
Myocardial infarction	2.272	1.247–4.141	0.007	0.695	0.362–1.335	0.275
Congestive heart failure	3.842	2.723–5.422	<0.001	1.209	0.814–1.796	0.347
Peripheral vascular disease	2.896	2.154–3.894	<0.001	1.097	0.785–1.535	0.587
Cerebrovascular disease	3.201	2.339–4.382	<0.001	1.031	0.705–1.507	0.876
Dementia	3.364	2.387–4.741	<0.001	0.771	0.510–1.164	0.216
Chronic pulmonary disease	1.715	1.304–2.258	<0.001	1.087	0.806–1.466	0.585
Rheumatologic disease	1.759	1.156–2.675	0.008	1.229	0.790–1.912	0.362
Peptic ulcer disease	1.839	1.400–2.415	<0.001	1.032	0.765–1.391	0.836
Mild liver disease	2.283	1.737–3.001	<0.001	0.985	0.718–1.352	0.926
Diabetes without chronic complications	3.808	2.898–5.005	<0.001	1.294	0.919–1.821	0.14-
Diabetes with chronic complications	5.137	3.721–7.093	<0.001	**1.811**	**1.241**–**2.642**	**0.002**
Paralysis (hemiplegia or paraplegia)	3.219	1.754–5.907	<0.001	1.102	0.566–2.146	0.776
Renal disease	4.281	2.540–7.215	<0.001	1.434	0.813–2.531	0.213
Malignancy	3.132	2.203–4.453	<0.001	1.406	0.946–2.091	0.092
Moderate or severe liver disease	2.876	0.675–12.248	0.153	1.364	0.301–6.188	0.687
Metastatic solid tumor	3.821	1.627–8.973	0.002	1.810	0.702–4.665	0.219
Acquired immune deficiency syndrome	0.000	0.000–999.999	0.976	0.000	0.000–999.999	0.983

Text in bold means a statistically significant difference in the multivariate analysis.

ICU, intensive care unit; OR, odds ratio; CI, confidence interval.

### Significant factors affecting duration of hospital stay

Age and underlying diseases—except rheumatologic disease, moderate or severe liver diseases, and acquired immune deficiency syndrome—were found to be significant factors that influenced the duration of hospital stay in univariate analysis. According to the multivariate analysis results, the duration of hospital stay was significantly lengthened when patients were old (*β* coefficient, 0.065; *P*<0.001), had concomitant cerebrovascular disease (*β* coefficient, 1.612; *P* = 0.002), chronic pulmonary disease (*β* coefficient, 0.831; *P* = 0.004), or paralysis (*β* coefficient, 3.285; *P* = 0.002) (Table 4).

**Table 4:** Significant factors for admission duration.

Parameters	Univariate analysis		Multivariate analysis	
	*β* coefficient	*P*-value	*β* coefficient	*P*-value
**Age (years)**	0.097	<0.001	**0.065**	**<0.001**
**Sex (female)**	0.437	0.125	0.219	0.440
**Prevalence of underlying disease**				
Myocardial infarction	2.790	0.001	0.081	0.929
Congestive heart failure	3.746	<0.001	0.686	0.259
Peripheral vascular disease	2.075	<0.001	-0.556	0.205
Cerebrovascular disease	4.345	<0.001	**1.612**	**0.002**
Dementia	4.123	<0.001	0.308	0.611
Chronic pulmonary disease	1.667	<0.001	**0.831**	**0.004**
Rheumatologic disease	0.835	0.118	-0.462	0.395
Peptic ulcer disease	1.540	<0.001	0.172	0.584
Mild liver disease	2.031	<0.001	0.284	0.392
Diabetes without chronic complications	2.932	<0.001	0.577	0.152
Diabetes with chronic complications	3.457	<0.001	0.554	0.365
Paralysis (hemiplegia or paraplegia)	6.738	<0.001	**3.285**	**0.002**
Renal disease	4.139	<0.001	1.651	0.093
Malignancy	2.940	<0.001	1.038	0.069
Moderate or severe liver disease	-0.392	0.870	-2.589	0.276
Metastatic solid tumor	3.910	0.013	1.521	0.347
Acquired immune deficiency syndrome	-5.984	0.194	-7.954	0.081

Text in bold means a statistically significant difference in the multivariate analysis.

### Significant factors influencing total hospital cost

We determined that the significant factors that contributed to total hospital cost in Korean Won such as age, sex, and underlying diseases—except for moderate or severe liver diseases and acquired immune deficiency syndrome—impacted total hospital costs in univariate analysis. In multivariate analysis, old age (*β* coefficient, 60,885; *P*<0.001) and being male (female *β* coefficient, -893,143; *P*<0.001) were risk factors for increased total hospital cost. Congestive heart failure (*β* coefficient, 1,165,577; *P*<0.001), chronic pulmonary disease (*β* coefficient, 453,242; *P* = 0.004), diabetes without chronic complications (1,026,449; *P*<0.001), renal disease (*β* coefficient, 1,789,702; *P* = 0.001), malignancy (*β* coefficient, 922,209; *P* = 0.003), and metastatic solid tumor (*β* coefficient, 2,582,275; *P* = 0.004) were significant factors that increased hospital cost (Table 5).

**Table 5:** Significant factors for the total hospital cost.

Parameters	Univariate analysis		Multivariate analysis	
	*β* coefficient	*P*-value	*β* coefficient	*P*-value
**Age (years)**	74,781	<0.001	**60,885**	**<0.001**
**Sex (female)**	-693,714	<0.001	**-893,143**	**<0.001**
**Prevalence of underlying disease**				
Myocardial infarction	1,374,958	0.005	-864,257	0.083
Congestive heart failure	3,144,068	<0.001	**1,165,577**	**<0.001**
Peripheral vascular disease	1,740,131	<0.001	30,392	0.900
Cerebrovascular disease	1,953,548	<0.001	78,942	0.787
Dementia	2,087,732	<0.001	-606,724	0.069
Chronic pulmonary disease	1,008,164	<0.001	**453,242**	**0.004**
Rheumatologic disease	1,022,204	<0.001	140,774	0.638
Peptic ulcer disease	926,043	<0.001	-2451	0.989
Mild liver disease	1,285,736	<0.001	-262,483	0.151
Diabetes without chronic complications	2,559,127	<0.001	**1,026,449**	**<0.001**
Diabetes with chronic complications	2,713,274	<0.001	408,814	0.225
Paralysis (hemiplegia or paraplegia)	1,499,017	0.009	-628,917	0.287
Renal disease	3,860,969	<0.001	**1,789,702**	**0.001**
Malignancy	2,524,916	<0.001	**922,209**	**0.003**
Moderate or severe liver disease	903,871	0.500	-938,758	0.473
Metastatic solid tumor	4,560,340	<0.001	**2,582,275**	**0.004**
Acquired immune deficiency syndrome	-18,179	0.994	-1,263,459	0.615

Text in bold means a statistically significant difference in the multivariate analysis.

## Discussion

The prognosis of COVID-19 was independently affected by underlying diseases, even after adjusting for age, sex, and other medical conditions. Underlying diseases are frequent in old age. In addition, some underlying diseases are significantly associated with other underlying diseases (for example, cerebrovascular disease and dementia; myocardial infarction and peripheral vascular disease). Therefore, a detailed analysis with adjustments was needed to determine whether underlying diseases were independent risk factors for the severity of COVID-19 presentation. To the best of our knowledge, this is the first study to confirm that underlying diseases are independent risk factors for poor prognosis of COVID-19, after adjusting for age, sex, and other chronic conditions. In addition, we also found that the prognosis and outcomes of patients with COVID-19 varied according to the type of underlying diseases that the patients had.

Underlying renal disease had the greatest effect on COVID-19 prognosis, especially death (OR, 2.299) and increased hospital cost. In renal diseases, the disturbance of toll-like receptors and alterations in the immune system contribute to immunodepression in those with said affliction [9]; such patients are more susceptible to infection and have difficulty recovering when infected. In addition, impaired renal function serves as a contraindication to the administration of pharmacologic treatment against COVID-19; or the dose of medication needs to be adjusted based on the renal function; this can lead to suboptimal treatment outcomes.

Diabetes is the next underlying disease that was found to have a significant effect on death (OR, 1.518–1.821), total hospital cost, and ICU admission of patients with COVID-19. A recent study reported that patients with diabetes are highly susceptible to COVID-19, and various mechanisms were proposed [10]. Diabetes can cause multiple metabolic and vascular abnormalities, affecting the patient’s response to pathogens [11]. Uncontrolled hyperglycemia is a risk factor for infections (e.g., pneumonia and influenza) and subsequent poor outcomes; therefore, strict glycemic control is critical for infection control [11–13]. Interestingly, our findings that diabetes increases the risk for poor prognosis in COVID-19 is consistent with similar previous studies in other coronavirus infections such as middle east respiratory syndrome and severe acute respiratory syndrome [12,14–16].

Congestive heart failure was also found to be a significant predictive factor for poor clinical outcomes in COVID-19, including death (OR, 1.724) and increased total hospital cost. Patients with congestive heart failure frequently present with dyspnea, cough, edema, and pleural effusion. These symptoms are similar to those of COVID-19; hence, symptoms from heart failure, especially dyspnea, can be aggravated in the presence of that infection. In addition, congestive heart failure is associated with an increase in endotoxins and cytokines [17] that can trigger the cytokine storm observed in COVID-19 that can prove fatal. Therefore, COVID-19-infected patients with congestive heart failure should be carefully monitored.

Dementia is another significant factor associated with death (OR, 1.598). Our findings are in keeping with those of studies on dementia being associated with prognosis in COVID-19; one such study reported how the presence of the *ApoE e4e4* allele—the gene associated with dementia—puts a patient at risk for severe COVID-19 [18,19]. Also, patients with dementia might be uncooperative in terms of expelling sputum, allowing for monitoring of vital signs, maintaining oxygen support, and even the medication intake. These factors may lead to poor outcomes of COVID-19 in patients with dementia.

Malignancy was revealed to be one of the important risk factors for mortality (OR, 1.520) and increased hospital cost. Moreover, metastasis from solid tumors was also a risk factor affecting those outcomes. Since malignancy alters cell-mediated immunity, patients with cancer—especially those in the advanced stages—become immunocompromised, and hence more susceptible to infections, including those of viral etiology [20,21]. In addition, immunosuppressive therapy used in cancer treatment may also contribute to failure of treatment for COVID-19.

Cerebrovascular disease and paralysis (hemiplegia or paraplegia) were revealed to be risk factors for longer hospital stay. In COVID-19, the route of infection is usually via the respiratory tract, and the cause of the death is usually a respiratory problem [22]. Patients with cerebrovascular disease or paralysis might be difficult to manage in the setting of COVID-19, as they might have problems in complying with management, including the expectoration of sputum. This may delay extubation and discharge.

Chronic pulmonary disease was also a significant factor found to contribute to increased hospital cost and prolonged hospital stay. COVID-19 is a respiratory infection, and therefore, the fact that prognosis of COVID-19 is poor in patients with chronic pulmonary disease is quite natural. However, the impact of chronic pulmonary disease on the COVID-19 were less significant compared to those of other underlying diseases. The prevalence of chronic pulmonary disease in this study was excessively high (44.6%). We followed the definition of chronic pulmonary disease as suggested in the HIRA guidelines; however, some diagnostic codes for chronic pulmonary disease in our data set might represent temporary pulmonary disease. The relatively high prevalence of chronic pulmonary disease in our study may have decreased the impact of that on the prognosis of patients with COVID-19. Further studies with a different definition of chronic pulmonary disease are needed to define its effects on the prognosis of COVID-19.

Unlike the other underlying diseases, acquired immune deficiency syndrome and moderate or severe liver disease ‎did not show any significant effects on prognosis after adjustment. However, the number of patients with acquired immune deficiency syndrome (n = 7) and moderate or severe liver disease (n = 26) included in this study was too small to adequately analyze their effect on the prognosis of COVID-19. To the best of our knowledge, there are only a few studies investigating the relationship between acquired immune deficiency syndrome and COVID-19. One case series reported that the mortality rate was not high (as 5 of 5 survived) and that only 1% of patients with COVID-19 who required hospitalization had acquired immune deficiency syndrome [23]. Further research on the relationship between acquired immune deficiency syndrome and COVID-19 is needed.

As expected, age was the single most important risk factor for increased hospital cost, prolonged hospital stay, ICU admission, and mortality in this study. This finding is consistent with those of previous studies [24–28]. We also found that the male sex was also an important risk factor in the prognosis of COVID-19. Although several studies have revealed sex disparities in the prevalence and prognosis of COVID-19 [26,29,30], a recent review reported that mortality was higher in males than in females, similar to the results of this study. The differences in angiotensin converting enzyme 2 and transmembrane serine protease 2 regulation and immune response to viral infections between males and females are emerging as the potential mechanisms [29]. The cell associated forms of the above two molecules are known to be important proteins in the intracellular invasion of severe acute respiratory syndrome coronavirus 2, and various studies have reported that sex hormones, such as estrogen, regulate these two proteins [31–35].

A strength of this study is its national coverage, which includes 7,590 patients with COVID-19. Also, to the best of our knowledge, this study is the first to reveal the variable effects of underlying diseases on the prognosis of patients with COVID-19.

However, there are also some limitations to this study. First, this study did not include important clinical data such as physical examination findings and laboratory findings that may have affected the results. Second, the diagnosis of underlying diseases was simply defined by diagnostic codes. Therefore, the prevalence of underlying disease was relatively high—especially chronic pulmonary disease—compared with that in previous studies [4]. South Korea has a national insurance system, and therefore, medical costs are relatively low. Given this, people in South Korea are admitted to the hospital for trivial symptoms such as mild cough or heartburn. This might explain why the prevalence of some underlying diseases, including chronic pulmonary and peptic ulcer diseases, seems to be higher than we expected in general population. Lastly, in the future, further studies with a larger sample size are needed to further define the effects of rare underlying diseases, including acquired immune deficiency syndrome, on COVID-19 prognosis.

## Conclusion

In conclusion, each underlying disease in this study had an independent variable effect on the clinical outcomes of COVID-19 after adjustments for age, sex, and other chronic conditions. Renal disease, diabetes, congestive heart failure, dementia, and malignancy proved to be risk factors for mortality. Cerebrovascular disease and paralysis prolonged duration of hospital stay. Chronic pulmonary disease increased total hospital cost and delayed the discharge date. In conclusion, patients with underlying diseases must be monitored more carefully because they are at a higher risk of poor outcomes while admitted for COVID-19.

## Supporting information

S1 Fig Odds ratio and 95% confidence interval for mortality during treatment for COVID-19 according to the type of underlying diseases. COVID-19, coronavirus disease; CHF, congestive heart failure; DEM, dementia; DM1, diabetes without chronic complications; DM2, diabetes with chronic complications; Ren, renal disease; Mal, malignancy.
(PPTX)

S1 Table ICD-10 coding algorithms for calculating the Charlson comorbidity index. ICD-10, international classification of diseases, 10^th^ Revision; AIDS, acquired immunodeficiency syndrome; HIV, human immunodeficiency virus.
(DOCX)

## Abbreviations

CI: confidence interval
COVID-19: coronavirus disease
HIRA: Health Insurance Review and Assessment Service
OR: odds ratio
