# Supplementary material for: Variable effects of underlying diseases on the prognosis of patients with COVID-19
Source: PLoS One. 2021 Jul 19;16(7):e0254258. doi: 10.1371/journal.pone.0254258 (PMC8289057; doi:10.1371/journal.pone.0254258)
Supplement: S1 Fig — COVID-19, coronavirus disease; CHF, congestive heart failure; DEM, dementia; DM1, diabetes without chronic complications; DM2, diabetes with chronic complications; Ren, renal disease; Mal, malignancy. (PPTX) [file pone.0254258.s001.pptx]

## Slide 1
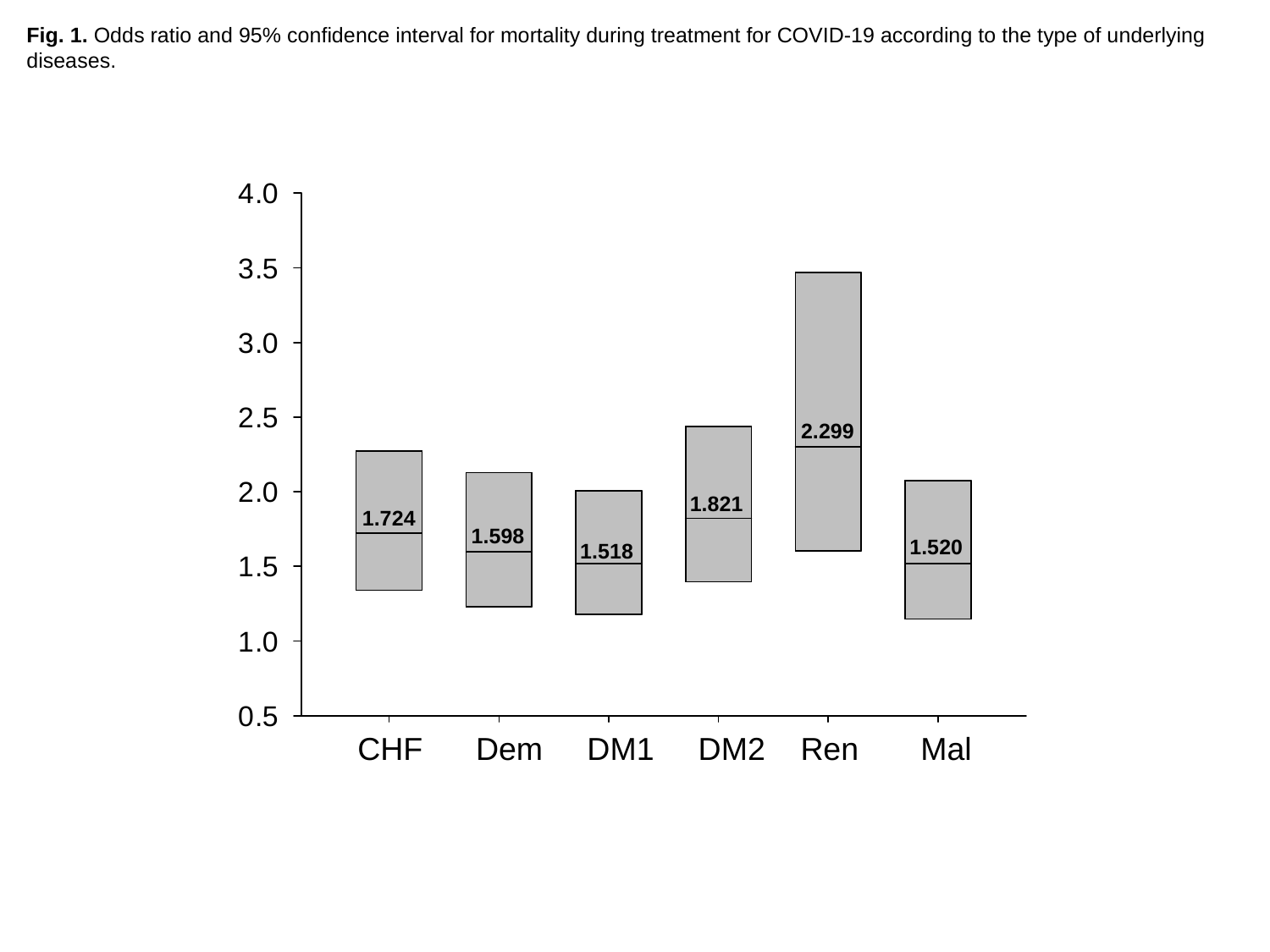

Fig. 1. Odds ratio and 95% confidence interval for mortality during treatment for COVID-19 according to the type of underlying diseases.
2.299
1.821
1.724
1.598
1.520
1.518
CHF Dem DM1 DM2 Ren Mal
